# A Lightweight Network with Domain Adaptation for Motor Imagery Recognition

**DOI:** 10.3390/e27010014

**Published:** 2024-12-27

**Authors:** Xinmin Ding, Zenghui Zhang, Kun Wang, Xiaolin Xiao, Minpeng Xu

**Affiliations:** 1 Academy of Medical Engineering and Translational Medicine, Tianjin University, Tianjin 300384, China; hxyy_ding@163.com (X.D.); 18205481257@163.com (Z.Z.); flora_wk@tju.edu.cn (K.W.); xiaoxiao0@tju.edu.cn (X.X.); 2 West China Tianfu Hospital, Sichuan University, Chengdu 610041, China; 3 Haihe Laboratory of Brain-Computer Interaction and Human-Machine Integration, Tianjin 300392, China

**Keywords:** BCI, motor imagery, transfer learning, convolutional neural network

## Abstract

Brain–computer interfaces (BCI) are an effective tool for recognizing motor imagery and have been widely applied in the motor control and assistive operation domains. However, traditional intention-recognition methods face several challenges, such as prolonged training times and limited cross-subject adaptability, which restrict their practical application. This paper proposes an innovative method that combines a lightweight convolutional neural network (CNN) with domain adaptation. A lightweight feature extraction module is designed to extract key features from both the source and target domains, effectively reducing the model’s parameters and improving the real-time performance and computational efficiency. To address differences in sample distributions, a domain adaptation strategy is introduced to optimize the feature alignment. Furthermore, domain adversarial training is employed to promote the learning of domain-invariant features, significantly enhancing the model’s cross-subject generalization ability. The proposed method was evaluated on an fNIRS motor imagery dataset, achieving an average accuracy of 87.76% in a three-class classification task. Additionally, lightweight experiments were conducted from two perspectives: model structure optimization and data feature selection. The results demonstrated the potential advantages of this method for practical applications in motor imagery recognition systems.

## 1. Introduction

Brain–computer interfaces (BCIs) represent a promising emerging field of research [1,2]. They have the potential to facilitate communication between the brain and external devices by encoding the characteristics of the brain’s response to specific operational tasks and translating them into instructions that can be understood by an external device [3]. In a motor imagery (MI) task, the brain generates spontaneous neural signals when a subject imagines an action occurring without performing it [4,5]. These signals have been the subject of considerable research in the field of BCIs, as the cognitive executive state evoked by MI has the potential to provide insights for the resolution of human–computer interaction issues in a multitude of ways [6,7]. It is anticipated that motor imagery recognition will assist patients with paralysis in performing basic activities of daily living [8]. Furthermore, the combination of motor imagery recognition with artificial intelligence algorithms is anticipated to facilitate investigation of the human brain’s mechanism of issuing intentional commands, thereby advancing the development of brain-inspired algorithms [9].

The most commonly employed techniques for the acquisition of brain signals in BCI systems are electroencephalography (EEG) [10,11,12], functional magnetic resonance imaging (fMRI), and functional near-infrared spectroscopy (fNIRS) [13]. Among the techniques is functional near-infrared spectroscopy (fNIRS), a non-invasive functional brain imaging technique that employs light with a wavelength of 600–900 nm to quantify alterations in cortical blood oxygen saturation and investigate functional brain activity [14]. In recent years, electroencephalography (EEG) and functional near-infrared spectroscopy (fNIRS) have become a widely used tools for brain function testing. This is due to the advantages they offer in terms of portability and non-invasiveness. Furthermore, there is considerable potential for applications in the fields of motor control, medical rehabilitation, and assisted surgery [15].

With the rapid development of deep learning, researchers have applied these techniques to MI-based intention recognition, significantly improving feature extraction and classification performance. For example, Liu et al. [16] compared the advantages and disadvantages of machine learning and deep learning methods, and found that a long short-term memory network (LSTM) achieved an accuracy of 82.86%, which was significantly better than the classification effect of the support vector machine (SVM). To avoid the impact of manual feature selection on performance, Trakoolwilaiwan et al. [17] applied a CNN as an automatic feature extractor and classifier and achieved an accuracy of 92.68% under a triple classification finger tapping task. Similarly, Hamid et al. [18] proposed a BCI framework for motion execution classification of fNIRS signals, and they also introduced a bidirectional long short-term memory network (BiLSTM) and various machine learning methods. The experimental results demonstrated that the performance of fNIRS-BCI can be significantly improved using deep learning methods.

Despite the promising results of deep learning models in MI tasks, their applications face two major challenges. First, deep learning models typically require a large number of parameters and high-quality labeled data. Second, insufficient data often leads to performance degradation. Transfer learning is regarded as an effective solution to these issues, as it transfers knowledge from a source domain to a target domain, reducing the demand for training data and accelerating model convergence. This is particularly beneficial in scenarios with limited labeled data.

In the context of EEG-based motor imagery recognition, Huang et al. [19] employed a data enhancement technique to augment their convolutional neural network (CNN) model, thereby facilitating the acquisition of more comprehensive data features for motion image classification. On the other hand, as principal component analysis (PCA) can enhance the computational velocity of a model by compressing the effective data features, Cheng et al. [20] put forth a model that integrates PCA with deep belief networks. Furthermore, given the high temporal resolution of EEG data, Dai et al. [21] constructed a multiscale temporal convolutional neural network for classification, utilizing convolutional kernels of varying sizes. Furthermore, Li et al. [22] constructed a DWT-LSTM model for motor imagery classification, which employed a long short-term memory network integrated with a wavelet transform in addition to a CNN-based model. While EEG signals offer significant advantages for motion image classification, they lack the requisite spatial information to effectively monitor changes in brain states. This is due to the fact that electroencephalogram (EEG) signals primarily monitor the activity of neurons on the scalp over time, and therefore do not provide the necessary spatial resolution. Furthermore, EEG signals are vulnerable to environmental noise, which presents a significant challenge for their utilization in brain–computer interface tasks [23]. In light of these constraints, the fNIRS technique has emerged as a promising avenue for further research in this field [24].

With respect to fNIRS, Almulla et al. [25] investigated the link between fNIRS and behavioral observation, and demonstrated that fNIRS has a promising future in motor imagery tasks and rehabilitation therapy. Wang et al. [26] combined EEG with FNIRS and proposed a weighted model that weights the classification effects of the two signals to construct a fused cognitive model for judging motor imagery instructions. Qin et al. [27] proposed an end-to-end model using FNIRS to combine temporal convolutional neural networks and spatial convolutional networks, and fused the two features for motor imagery classification. In addition, the current transfer learning models for fNIRS signal recognition mainly use direct sample-based migration [28,29] and model-based fine-tuning [30,31]. Direct migration applies the trained model in the training set directly to test individuals. For example, Wang et al. [28] conducted leave-one-subject cross-validation, which is regarded as direct migration. Fine-tuning, on the other hand, involves retraining and fine-tuning a pre-trained neural network model using labeled data from the target domain [30]. Yao et al. [31] proposed a modified inception-ResNet model by fixating the lower layers of the model and fine-tuning the higher layers in order to improve the cross-subject decoding accuracy for mental tasks and effectively reduce the model training time. Transfer learning methods based on samples and models obtained better results in the fNIRS classification task [32]. However, it is worth noting that these two methods have high requirements for target domain data, and the migration effect is poor when the sample distributions of the target domain and the source domain are highly different.

However, when there are significant distribution differences between the source and target domains, the effectiveness of conventional transfer learning methods is limited. Additionally, fNIRS signals are influenced by individual differences, acquisition noise, and motion artifacts, which result in signal non-stationarity and domain bias. These factors further increase the difficulty of transfer learning.

In light of the current trend in BCI technology towards efficiency, this paper proposes a feature-based domain adaptation method to address cross-subject domain discrepancies in fNIRS signals, enabling robust and efficient cross-domain recognition. Specifically, a lightweight convolutional feature extraction module is introduced to retain critical signal features, while reducing model complexity and runtime, paving the way for real-time BCI applications. Then, a distribution alignment module is employed to reduce inter-domain discrepancies in the feature representation layer. Combined with domain adversarial training, this approach effectively learns domain-invariant features, enhancing cross-subject generalization. By leveraging the unique characteristics of fNIRS signals, the model focuses on addressing the high variability across subjects, further improving the adaptability. In this paper, we evaluated the model performance on a fNIRS motor imagery dataset and conducted lightweight model experiments.

## 2. Methods

**Figure 1:**
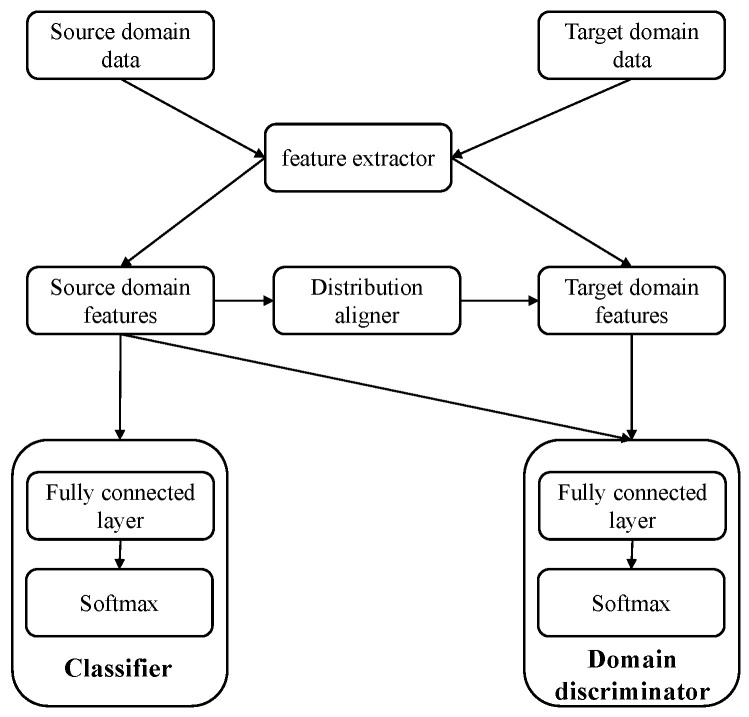
Motor imagery recognition model based on transfer learning.

In this paper, we propose a migration learning model based on a lightweight convolutional neural network and domain adaption (LCNN-DA), the model framework is shown in Figure 1.

The LCNN-DA consists of four main components: a feature extractor, distribution aligner, domain discriminator, and classifier. First, the feature extractor is used to refine and extract the key features of each domain. Second, the distribution aligner uses maximum mean discrepancy (MMD) [33], which is used to address the covariate bias caused by the change in edge distribution with the data domain. The domain discriminator is used to discriminate the domain and is trained against the feature extractor to learn domain-invariant features. Finally, the recognition task is accomplished by a classifier.

### 2.1. Feature Extractor

**Figure 2:**
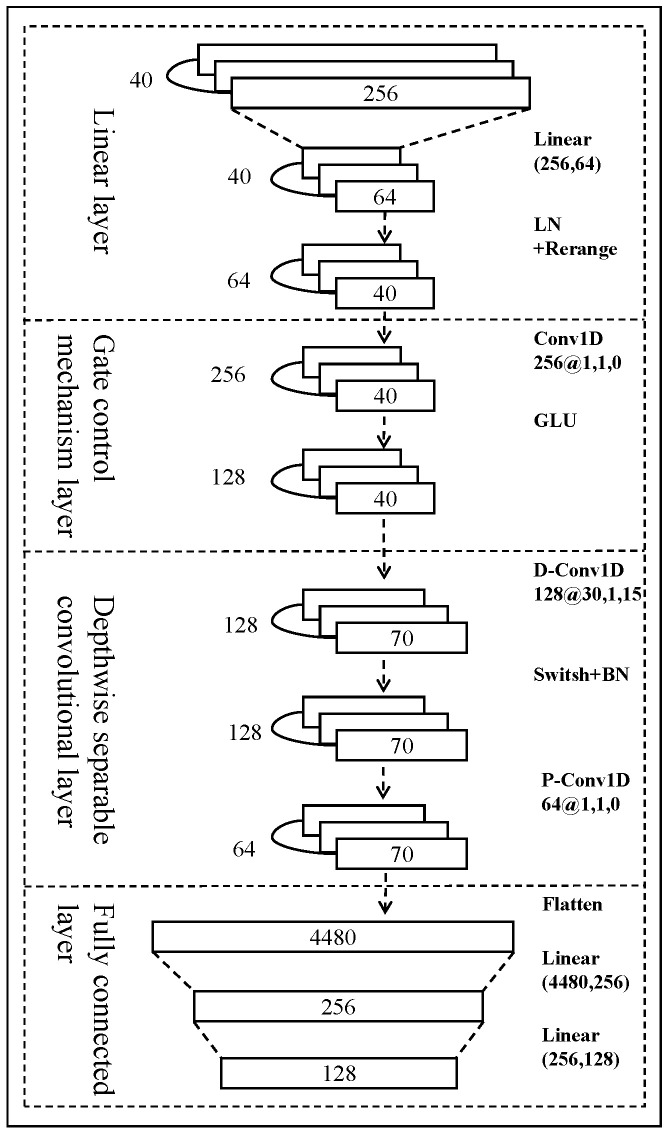
Feature extractor network architecture (convolution kernel parameters are expressed as number of kernel @ length of kernels, step size, number of fillers).

In fNIRS signal recognition, deep learning methods perform better compared to machine learning methods, but their huge number of parameters leads to huge computational costs and insufficient real-time performance. As a result, this paper proposes a lightweight feature extractor based on depth-separable convolution, which mainly includes a gating mechanism, depth-separable convolution, batch normalization, and fully connected layer, as shown in Figure 2.

The first layer of the network structure consists of a linear layer, LayerNorm (LN), and rearrangement: the linear layer is used for dimensionality reduction; the layer normalization stabilizes the input distribution in a suitable range; and the rearrangement is used for the subsequent processing. The second layer, a gating mechanism, consists of a pointwise convolution and a gated linear unit (GLU). A GLU is a gating mechanism in CNNs that makes it easier to propagate gradients and that is less likely to cause gradient vanishing or gradient explosion than the gated recurrent units of recurrent neural networks [34]. This structure is able to retain information strictly at the temporal position during temporal data processing, which improves the performance. Moreover, it can accelerate the speed of temporal data operations through a parallel processing structure, which is more efficient compared to a RNN. The third layer mainly adopts depthwise separable convolution, which consists of two parts: depthwise convolution and point-by-point convolution. The former is a one-dimensional (1D) channel-by-channel convolution layer, and the shape of the signal after channel-by-channel convolution is (128, 70). Batch normalization (BN) is deployed after the convolution to assist in training the deep models, and the Swish activation function is used to prevent gradient vanishing during slow training. The subsequent point-by-point convolution part includes a one-dimensional point-by-point convolution layer, dropout layer, and spread layer. The shape of the signal after point-by-point convolution is (64, 70). Compared with traditional convolution operations, depthwise separable convolution has fewer parameters and lower computational costs, which helps to achieve lightweight models [35]. The fourth layer, consisting of two fully-connected layers, is followed by a dropout layer with a parameter of 0.5 after each fully-connected layer, to prevent overfitting, improve the training speed, and enhance the generalization ability of the neural network model. After the feature extractor, the final features with 128 dimensions are obtained.

### 2.2. Distribution Aligner

The distribution aligner first computes the distances of the feature distributions in the source and target domains, and then learns by stochastic gradient descent, in order to make the two domains as similar as possible. MMD is a kernel learning method that measures the distance between two distributions in a regenerated Hilbert space [33]. It is relatively robust in recognizing differences between distributions and does not require making distributional assumptions about the data, making it a commonly used distance difference measure in transfer learning [36,37].

The MMD squared distance between two random variables is shown in the following equation:

(1) $$L_{\mathrm{MMD}}={∥\frac{\sum_{x_{s}\in x_{s}}Ø\left(x_{s}\right)}{\left|X_{S}\right|}-\frac{\sum_{x_{t}\in x_{T}}Ø\left(x_{t}\right)}{\left|X_{T}\right|}∥}^{2}$$

where ϕ is the kernel function for mapping the original variables to the regenerated kernel Hilbert space, and $Ø\left(x_{s}\right)$ and $Ø\left(x_{t}\right)$ are the representations of the random variables $x_{S}$ and $x_{T}$ in the feature space, respectively. It is worth noting that the distribution distances need to be added to the overall objective function to align the domain feature distributions.

### 2.3. Domain Discriminator

The domain discriminator is used to distinguish between features generated in different domains, and its generated domain discrimination loss portrays the difference in the edge distribution of the two data domains. Importantly, the domain discriminator is trained adversarially to the feature extractor [38]. Specifically, the domain discriminator is trained to perform domain discrimination between source and target features, while the goal of the feature extractor is to try to deceive the discriminator by attempting to generate indistinguishable representations for the source and target domains. This adversarial training effectively learns domain-invariant features such that classifiers trained in the source domain generalize well on the target domain [39]. The domain discriminator in this study consists of a fully connected layer and a Softmax function. The Softmax function receives the vector output from the fully connected layer as input, and converts each dimension into a certain real number in the interval (0, 1) for classification. For input *x*, the probability formula is

(2) $$p\left(x=i\right)=\mathrm{Softmax}\left(x_{i}\right)=\frac{e^{w_{i}x_{i}}}{\sum_{j=1}^{k}e^{w_{j}x_{j}}}$$

where *p* denotes *x* is the category *i* probability of the trained domain discriminator with a classification probability of 53.2%; *w* denotes the weight value; and *k* denotes the total number of categories. In this way, the probability distribution of each category can be obtained for the multiclassification problem of the domain discriminator. Thus, the domain discriminator resembles a binary classification task with the domain discrimination loss as follows:

(3) $$L_{\mathrm{domin}}=-\sum_{j=0}^{J}d_{j}\log d_{j}^{\prime }$$

where *J* represents the domain label, belonging to [0,1], $d_{j}$ represents the true domain label, and $d_{j}^{\prime }$ represents the predictive domain labeling. In particular, the domain discrimination loss is added to the overall objective function to ensure that the model can generate domain-invariant features.

### 2.4. Classifiers

The classifier, like the domain discriminator, consists of a fully connected layer and a Softmax function. In this case, the number of neurons in the fully connected layer is 3 and the Softmax function is used to triple categorize the outputs of the left hand, right hand, and feet. The loss function in the classifier is used to guide the model to learn the correct classification task with the following formula:

(4) $$L_{\mathrm{cls}}=-\frac{1}{n}\sum_{i=1}^{n}y_{i}\log P_{\theta }\left({\bar{y}}_{i}|x_{i}\right)$$

where *n* is the sample size; $y_{i}$ represents the true label; and $P_{\theta }\left({\bar{y}}_{i}|x_{i}\right)$ is the probability of the category predicted by the model.

### 2.5. Objective Function

The pairwise classification loss, domain distribution distance, and domain discrimination loss are integrated in an objective loss function, as shown in the following equation:

(5) $$L_{all}=L_{cls}+\lambda \left(L_{MMD}+L_{domin}\right)$$

Since the distribution aligner and the domain discriminator are two domain adaptive modules, in this paper, we designed a domain adaptation balancing factor λ to control their contributions. In the model, the λ is set to 0.35, which allows the objective function to be weighted primarily toward classification with sufficient regularization for domain feature adaptation.

## 3. Experiments

### 3.1. Datasets

This paper used the publicly available dataset [29], which contains fNIRS data from 30 participants. According to the International 10–20 system, fNIRS photodiodes were placed in the left hemisphere and right hemisphere motor cortex regions, and a total of 20 channels of data were acquired.

**Figure 3:**
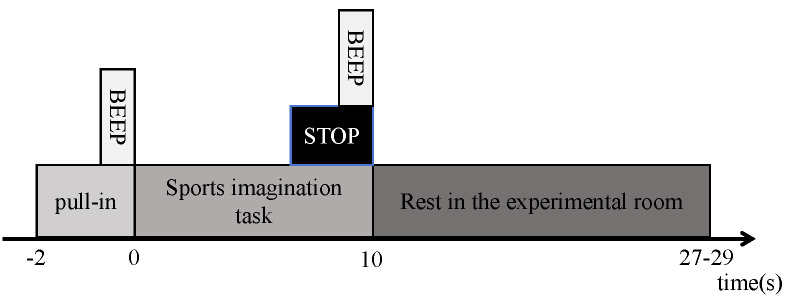
Experimental paradigm.

The experiment used a typical motor imagery paradigm, as shown in Figure 3. Each trial consisted of an introductory period (2 s) and a task period (10 s): the experiment started with a beep tone; then, participants were asked to perform a motor imagery task on the display: a right-hand finger tap, a left-hand finger tap, or a foot tap; at the end of the task, the display showed “STOP” with a beep tone; after a rest period of 17–19 s, the next trial started. Participants were asked to complete three types of motor imagery tasks, each of which was repeated 25 times in a randomized order.

### 3.2. Preprocessing

The inherent noise in the original NIR signal would have directly affected the feature extraction efficiency and thus the classification performance, so the data were first preprocessed. Usually, fNIRS data preprocessing includes the modified Beer–Lambert law (MBLL) [40], filtering, segmentation, and baseline correction. Based on the relationship between light intensity and medium concentration as light passes through a transparent medium, the MBLL allows for the conversion of light intensity signals captured by the instrument into brain blood oxygenation activity. Specifically, the change in optical density at time period Δt(ΔOD) is converted to the change in concentration of oxyhemoglobin (HbO) and deoxyhemoglobin (HbR) due to near-infrared light absorption, which is calculated as follows:

(6) $$\left[\begin{array}{c} \Delta HbO\\ \Delta HbR \end{array}\right]=\frac{1}{d}{\left[\begin{array}{cc} \varepsilon HbO(\lambda_{1}) & \varepsilon HbR(\lambda_{1})\\ \varepsilon HbO(\lambda_{2}) & \varepsilon HbR(\lambda_{2}) \end{array}\right]}^{-1}\left[\begin{array}{c} \Delta OD\left(\Delta t,\lambda_{1}\right)/DPF\left(\lambda_{1}\right)\\ \Delta OD\left(\Delta t,\lambda_{2}\right)/DPF\left(\lambda_{2}\right) \end{array}\right]$$

where *d* is the distance between the light source and the detector, $\lambda_{1}$ and $\lambda_{2}$ are the different illumination wavelengths, DPF is the differential path length factor for the illumination wavelengths, and ε is the extinction coefficient of HbR and HbO.

A Butterworth filter is a linear phase filter with flat frequency response and distortion-free characteristics [41]. Considering the correlation between fNIRS signals and neuronal activation in cerebral hemodynamics as a non-stationary process, a third-order Butterworth filter (with a passband of 0.01–0.1 Hz) was used to preserve useful neural activity information within this range, while suppressing lower physiological noise (such as breathing and heart rate fluctuations) and higher motion or environmental noise. Subsequently, according to the experimental paradigm flow, the fNIRS signals were segmented, with each segment containing data from 2 s before the start of the task to 28 s after the start of the task, and baseline correction was applied to the data segments using −1 to 0 s before the start of the task as the baseline [29], which was calculated in the following equation:

(7) $$BC\left(x\right)=x-u_{0}$$

where *x* denotes the fNIRS signal, and $u_{0}$ denotes the baseline segment mean.

Finally, considering the delayed hemodynamic response [42], the baseline-corrected data segments were segmented again by intercepting the data segments between sampling points 20 and 276 with a window length of 256.

### 3.3. Migratory Learning Strategies and Migratory Learning Paradigms

In the process of migration learning, this paper used three migration learning strategies, direct transfer (DT), fine-tuning (FT), and domain adaptation, to adjust the model. Among them, DT refers to the direct migration of a model and parameters to a new individual. FT contains two steps in total, first, the model and parameters are migrated to the target domain, and in order for the model to learn the specific patterns and features of the target domain data, the model parameters need to be fine-tuned using the target domain data. Domain adaption is migration learning using the idea of domain alignment and domain adversarial learning.

Multi-source to single-target (MTS) and single-source to single-target (STS) approaches were compared in the selection of migration paradigms. In this case, MTS is a cross-subject experiment in which one test individual is selected sequentially and the rest are used as the training set. STS selects one test individual sequentially, selects individual data from the rest of the individuals in turn to be used as the training set, and then finally takes the average value.

### 3.4. Lightweight Modeling Experimental Setup

In this paper, model lightweighting experiments were conducted from two aspects: model structure optimization and data feature selection. In structure optimization, the commonly used convolution module was replaced by depth-separable convolution. In feature selection, the region of interest (ROI) related to the task was selected from the original data, and the model parameters were reduced by data dimensionality reduction. Table 1 shows the model parameters of this paper based on the literature [29] and channel spatial distribution information for the channel selection of ROIs related to motor imagery task.

### 3.5. Experimental Environment

In this paper, we built the model based on pytorch version 1.8.0 framework and used an NVIDIA GeForce GTX 1660 SUPER GPU to train the model, and the experimental hyperparameter details are shown in Table 2.

### 3.6. Evaluation Indicators

In this paper, cross quilt test evidence was used to evaluate the model. The evaluation metrics included accuracy, precision, recall, F1 value, and Kappa value. The formulas for the various evaluation metrics are given below:

(8) $$Accuracy=\frac{TP+TN}{TP+TN+FP+FN}$$

(9) $$Precision=\frac{TP}{TP+FP}$$

(10) $$Accuracy=\frac{TP+TN}{TP+TN+FP+FN}$$

(11) $$F1=\frac{2\times Precision\times Recall}{Precision+Recall}$$

(12) $$Kappa=\frac{Accuracy-P_{e}}{TP+FP}$$

(13) $$P_{e}=\frac{\left(TP+FP)\times (FN+TP)+(FN+TN)\times (FP+TN\right)}{N^{2}}$$

In the formula, TP, TN, FP, and FN represent the number of instances of true positives, true negatives, false positives, and false negatives, respectively.

In this paper, we also used three evaluation metrics, namely the number of parameters, the number of floating-point operations, and the speed of reasoning (FPS), to evaluate the lightweighting effort. Among them, the number of parameters is the number of parameters to be learned in the model, which is usually used to indicate the complexity of a model. The number of floating-point operations is the number of floating-point operations required in the model training or inference process, which is usually used to measure the computational complexity of a model and the resource consumption at runtime. In this case, the training process was to tune the model by optimizing the model parameters to minimize the loss function, while the inference process was to use the already trained model to predict or classify new data. The inference speed (frames per second, FPS) is the speed at which a model can process input data on a given hardware device, usually measured in terms of the number of images or data points processed per second, and is calculated as follows:

(14) $$FPS=\frac{N}{T}$$

where *N* represents the number of samples processed, and *T* represents the total time required to complete inference on these samples. FPS represents the number of samples that the model can process per second.

## 4. Experimental Results and Analysis

### 4.1. Experiments on Model Parameters

In the third layer of the feature extractor, the kernel length of the depthwise separable convolution significantly affected recognition performance. To optimize the feature extractor and ensure that the model achieved optimal performance before introducing domain adaptation methods, a systematic evaluation of different kernel lengths was conducted. The experimental results are summarized in Table 3. As shown in Table 3, the recognition rate initially increased and then decreased with the increase in kernel length. When the kernel length was 30, the recognition rate reached 79.69% ± 14.12%, with a parameter count of only 1.23 M, striking the best balance between performance and computational efficiency. In contrast, when the kernel length was too short (e.g., 10 or 20), the model was unable to capture sufficient task features, resulting in a decrease in recognition rates to 71.88% and 74.43%, respectively. Conversely, when the kernel length was too long (e.g., 50 or 60), the model captured excessive irrelevant information, leading to a performance drop (with recognition rates of 75.79% and 69.20%, respectively).

This phenomenon indicates that the selection of kernel length should consider both data characteristics and task requirements. A kernel that is too short may fail to capture enough feature information, while an excessively long kernel may introduce too much irrelevant noise, negatively impacting performance. Therefore, the optimal kernel length was 30, which not only improved the recognition performance but also enhanced the computational efficiency by limiting the parameter size. Moreover, the choice of kernel length should align with the temporal dependencies of the task. To effectively extract task-specific features, the kernel should cover a sufficient time window to capture key information, while avoiding the inclusion of redundant or irrelevant features. The analysis of experimental results suggests that kernel length plays a crucial tuning role in task design, and the optimized kernel length provides key guidance for achieving higher recognition rates and more efficient models.

### 4.2. Model Performance

**Figure 4:**
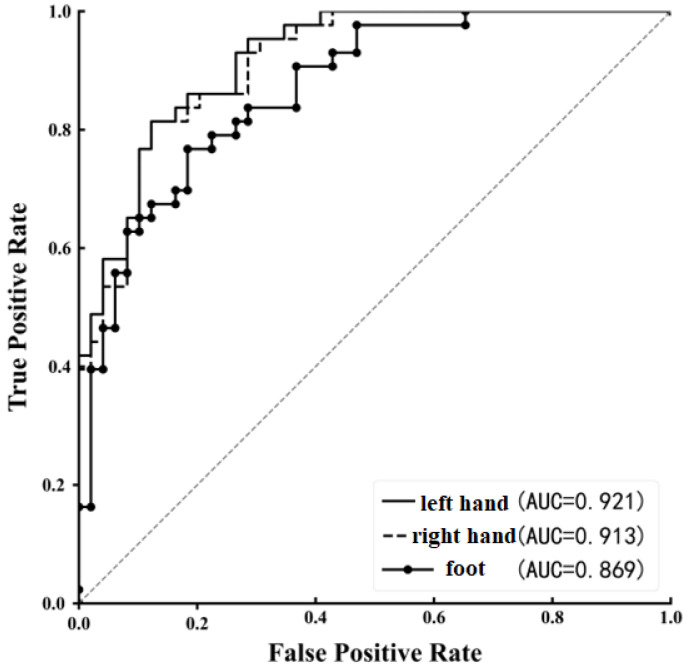
ROC curve of motor imagery classification task.

To comprehensively evaluate the performance of the LCNN-DA model, we plotted an ROC curve and calculated the area under the curve (AUC), as shown in Figure 4. The results clearly indicated that there were differences in classification performance across the three motor imagery tasks. Specifically, the AUC values for the left and right hand tasks were 0.921 and 0.913, respectively, both greater than 0.9, and significantly higher than the AUC value of 0.869 for the foot task. This suggests that the LCNN-DA model performed better in classifying the left and right hand tasks compared to the foot task. Furthermore, the ROC curve was close to the top-left corner, indicating that the LCNN-DA model achieved better classification performance for both positive and negative samples across the different classification thresholds.

**Figure 5:**
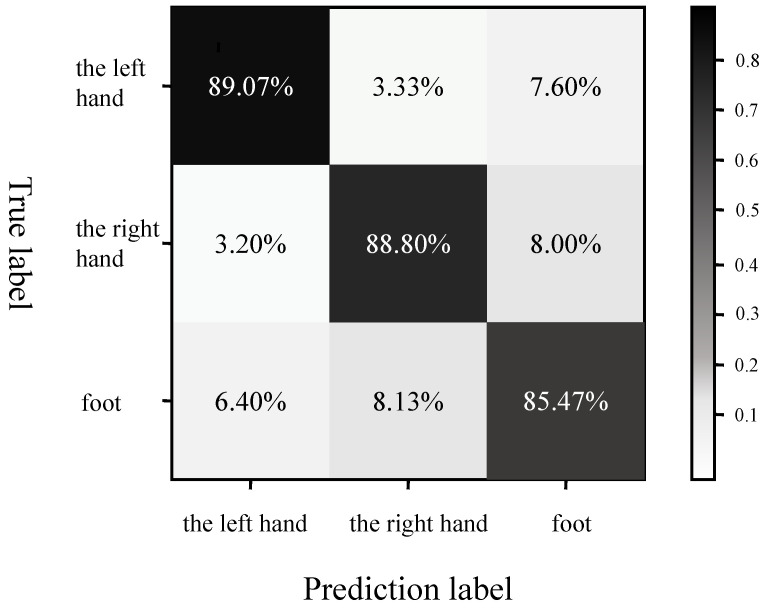
Confusion matrix for three-classification recognition.

The confusion matrix in Figure 5 further supports these findings, showing that the recognition rate for the left hand task was the highest at 89.07%. Although the recognition performance for the foot task was comparatively weaker, the rate of misclassification did not significantly increase, suggesting that the model was still capable of distinguishing between categories to a certain extent, despite the lower performance for the foot task. Taken together, the analysis of both the ROC curve and the confusion matrix leads to the conclusion that the LCNN-DA model demonstrated a strong classification performance in processing fNIRS motor imagery signals, particularly in the context of left-handed motor imagery tasks. However, there is still room for improvement in the classification performance for foot tasks.

### 4.3. Comparison with Other Transfer Learning Methods

**Figure 6:**
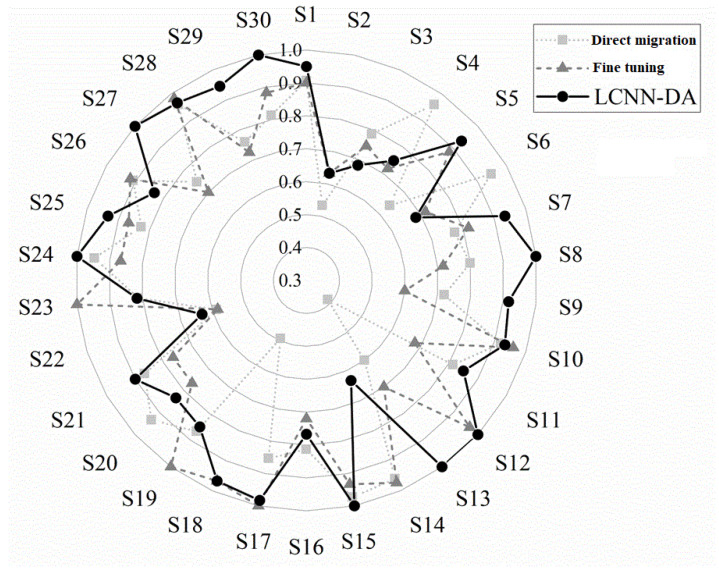
Classification accuracy of each subject with different transfer learning strategies (all subjects are represented as S1, S2, …S30).

In order to verify the proposed approach, we compared three migration learning methods, LCNN-DA, direct migration, and fine-tuning, where the feature extractor was frozen for the fine-tuning experiments. Figure 6 demonstrates the classification performance of individual subjects under the three strategies. From the classification accuracy of 30 subjects, the LCNN-DA model classified 23/30 subjects with an accuracy higher than 80% (including 18/30 higher than 90%). In particular, the performance of S7, S8, S9, S27, and S29 was greatly improved, with accuracy rates above 90%, while the accuracy rate of direct migration was lower than 80%. The accuracy varied greatly between subjects, e.g., S15 and S28 had a high classification accuracy, close to 100% under all three strategies, while S2 and S22 both had a classification accuracy close to 0.6.

Table 4 shows the impact of the three transfer learning strategies on the model performance metrics. As shown in the comparison, the direct migration strategy yielded relatively poor results, with all metrics lower than those achieved by the fine-tuned model. The fine-tuned classification layer better adapted to the target domain, thereby improving the classification performance. However, the most significant improvement occurred when the domain adaptation strategy was applied. The performance of the LCNN-DA model was notably enhanced, achieving an accuracy of 87.76%, an F1 score of 87.68%, and a Kappa value of 81.60%, representing an almost 8% improvement compared to direct migration. These results indicate that the LCNN-DA strategy not only significantly improved the accuracy of fNIRS motor imagery classification but also effectively handled fNIRS signals with high individual variability, thus demonstrating the importance and effectiveness of domain adaptation in such tasks.

### 4.4. Comparison of Transfer Learning Paradigms

**Figure 7:**
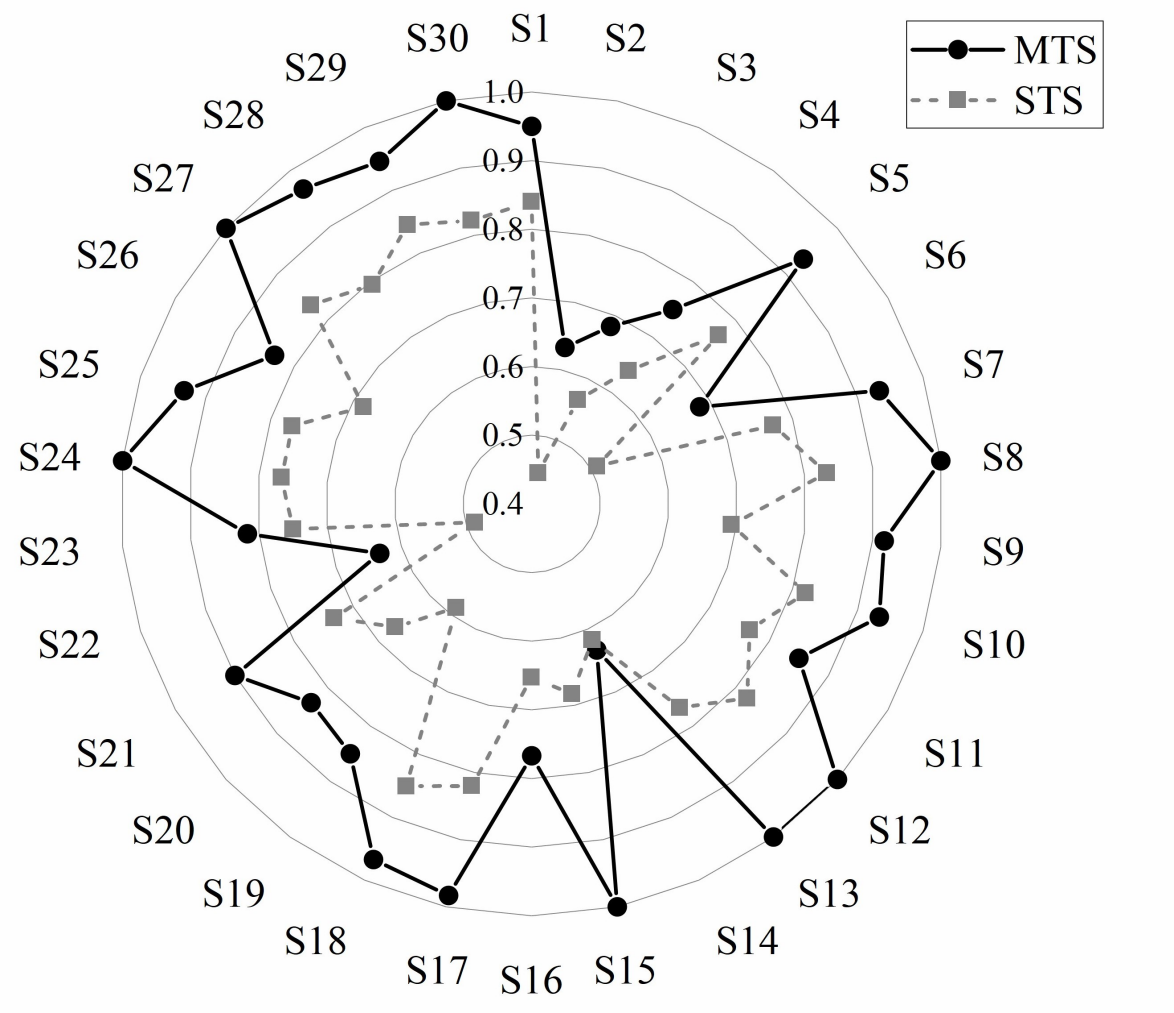
Classification accuracy of each subject with different transfer learning paradigms.

Figure 7 shows the classification accuracies of individual subjects under both migration paradigms. It can be seen that MTS was significantly higher than STS for all subjects, with the only exception being for S14, where very similar classification accuracies were achieved between MTS and STS, but both had very low accuracies of around 60%. In addition, some individuals had particularly low STS classification accuracies, such as S2 and S22, with less than 50% classification accuracy. Overall, the average classification accuracy of MTS (87.76%) was 15.75% higher than that of STS (72.01%), respectively. This may have been due to the fact that the source domain of MTS was a fusion of data from multiple remaining subjects, whereas STS only used data from a single subject as the source domain, and thus the model could not fully learn the features during the training period. This is consistent with the fact that deep models are data-driven, and the more training data there are, the higher the classification accuracy will naturally be.

### 4.5. Lightweight Modeling Study

Table 5 and Table 6 present the results of model lightweighting through structural optimization and feature selection methods. In terms of structural optimization, we compared the performance of models using traditional convolution and depthwise separable convolution (DSC). In terms of accuracy, the conventional convolution achieved an accuracy of 88.54%, while the depthwise separable convolution achieved 87.76%, with a difference of less than 1%. However, the application of DSC significantly reduced the model’s number of parameters (by 0.5 M) and the number of floating-point operations (by 20.39 M), while also improving the inference speed (from 859 samples/s to 907 samples/s). This indicates that depthwise separable convolution effectively reduced the computational burden, while maintaining high accuracy.

In the feature selection aspect, compared to experiments using the full dataset, models with a reduced channel dataset showed no significant drop in performance and, in some cases, still demonstrated relatively good results. Specifically, the accuracies of ROI A (77.41%), B (76.66%), and C (75.24%) remained above 60%, despite having less than half the channels of the full dataset, with the accuracy reduced by about 10%. Notably, ROI B, which used only one-fifth of the number of channels, significantly reduced the model parameters (by 0.706 M) while maintaining a high inference speed (1172 samples/s). This further demonstrates the effectiveness of feature selection in reducing model complexity and improving efficiency.

### 4.6. Comparison with Other Methods

Table 7 presents a comparison of the performance of the proposed model with recent methods in the literature (all from fNIRS datasets). Compared to traditional approaches, the LCNN-DA model demonstrated significant advantages. First, the study by Bak et al. [29] used support vector machines (SVM) based on ΔHbO/R features for classification, with feature selection based on manual feature extraction. Although the method achieved an accuracy of 70.40%, it relied heavily on manual feature extraction, which is computationally complex and prone to human biases. In contrast, Esfahani et al. [43] proposed a non-dominated sorting multi-objective genetic algorithm based on temporal features that effectively reduced computational costs and feature dimensions during feature selection. However, the final classification accuracy was only 70.80%, and the optimization process remained relatively complex, without significantly overcoming the accuracy limitations.

In Siddique et al.’s study [32], Bayesian neural networks (BNN) were employed, where Bayesian statistics were used to assign probability distributions to network weights. The model achieved an accuracy of 86.44%. However, it is important to note that this model was applied to a binary classification task involving unilateral finger tapping, which is inherently less complex than the three-class task used in this study, making a direct comparison difficult. Wang et al. [28] explored using Transformer networks for fNIRS signal classification. By optimizing spatial and channel-level feature representations of near-infrared spectral signals, they successfully improved the data utilization and network representation capabilities. However, their model achieved an accuracy of 78.22%, still falling short of the performance demonstrated by the LCNN-DA model.

In addition, Li et al. [44] introduced ID-CapsuleNet, which combined large kernel dilated convolutions with CapsuleNet to optimize hemodynamic feature extraction, achieving an accuracy of 75.78%. Wang et al. [45] presented fNIRSNet, which incorporated domain knowledge and reduced computational parameters while optimizing the model structure, with an accuracy of 75.47%. While both methods brought innovations in feature extraction and model simplification, their accuracy remained lower than that of LCNN-DA.

In summary, LCNN-DA outperformed all other methods, with an accuracy of 87.76%, clearly demonstrating superior classification performance in fNIRS motor imagery tasks. By incorporating more efficient feature extraction and deep learning models, LCNN-DA not only improved the accuracy but also optimized the computational efficiency, providing a more reliable solution for fNIRS signal decoding tasks.

In addition, our model had a training time of 0.070995 and a testing time of 0.013999, slightly higher than SVM and LDA. Compared to other non-transfer-learning deep learning and machine learning methods, our model has a lower time cost and can achieve similar or better model performance. The results indicate that our model achieves a good balance between efficiency and accuracy, with higher flexibility and lower cost. Meanwhile, compared to other models with high time costs, it is easier to deploy.

**Table 7:** Comparison with other research methods.

Reference	Method	Accuracy/%	Training Time (s)	Testing Time (s)
**Bak et al. [29]**	SVM	70.40 ± 18.40	0.060431	0.009844
**Esfahani et al. [43]**	LDA	70.80 ± 10.98	0.041124	0.007911
**Siddique et al. [32]**	BNN	86.44	0.064248	0.011220
**Wang et al. [28]**	fNIRS-T	78.22 ± 16.69	0.122503	0.034425
**Wang et al. [45]**	fNIRSNet	85.47 ± 17.61	0.115521	0.025427
**Li et al. [44]**	ID-CapsuleNet	83.78 ± 16.73	0.132045	0.041283
**This paper**	LCNN-DA	87.76 ± 12.23	0.070995	0.013999

## 5. Conclusions

In this paper, a motor imagery recognition method based on transfer learning was proposed to solve the problem of the large differences in the distribution of data from different individuals, which is prone to domain bias. First, the model was based on depth-separable convolution, and a feature extractor was designed to extract features from the source and target domains to satisfy real-time BCI applications. Then, MMD was used to align the feature distribution and learn domain-invariant features adaptively through adversarial domain adaption. Finally, classification recognition was performed by a classifier. The experimental results showed that the application of deep separable convolution reduced the model parameters from 1.73 M to 1.23 M, the number of parameters was again reduced to 0.71 M by means of feature selection, and the classification accuracy reached 76.66%. Compared with the migration strategy of direct migration and fine-tuning, the migration learning strategy proposed in this paper showed a certain degree of improvement in the recognition rate, and the average recognition rate reached 87.76%, which reflects that LCNN-DA could improve the model generalization performance. In summary, the method in this paper could effectively solve the problem of individual differences and large differences in data distributions, and the model has strong applicability, which can provide strong support for BCI-based operational intent recognition. However, real-time applications still face some challenges, such as the limitation of computational resources and further optimization of individual adaptive differences, which need to be further explored and solved in future research.

## Figures and Tables

**Table 1:** Channel selection for region of interest.

ROI	Corresponding Channel	Channel Number
A	1–4, 11–14	8
B	5, 6, 15, 16	4
C	4–7,14–17	8
D	8, 9, 18, 19	4
E	7–10, 17–20	8

**Table 2:** Experimental configuration.

Hyperparameter Name	(Be) Worth
initial learning rate	0.001
weight decay	0
dropout	0.5
batch iteration	120
batch Size	128
loss function	cross-entropy
optimizer	Adam

**Table 3:** Effect of convolution kernel length.

Kernel Length	Accuracy/%	Number of Parameters/M
10	71.88 ± 17.98	0.90
20	74.43 ± 16.63	1.06
30	79.69 ± 14.12	1.23
40	78.16 ± 13.52	1.39
50	75.79 ± 13.69	1.56
60	69.20 ± 11.29	1.73

**Table 4:** Comparison of different transfer learning strategies.

Method	Accuracy/%	Precision/%	Recall/%	F1/%	Kappa /%
**Direct migration**	79.69 ± 14.12	78.20 ± 15.18	76.76 ± 16.12	76.24 ± 16.68	65.13 ± 21.18
**fine-tuning**	82.31 ± 16.55	80.24 ± 15.09	81.56 ± 16.55	80.78 ± 16.98	75.33 ± 20.83
**LCNN-DA**	87.76 ± 12.23	88.67 ± 11.31	87.70 ± 12.23	87.68 ± 12.33	81.60 ± 18.34

**Table 5:** Effect of convolution neural network.

Structure	Accuracy/%	Number of Parameters/M	FLOPs/M	Inference Speed /(Sample/s)
**CNN**	88.54 ± 12.10	1.73	23.78	859
**DSC**	87.76 ± 12.23	1.23	3.39	907

**Table 6:** Effect of region of interest selection.

Feature Selection	Accuracy/%	Number of Parameters/M	FLOPs/M	Inference Speed /(Sample/s)
**ROIA**	77.41 ± 15.82	0.84	1.90	1047
**ROIB**	76.66 ± 13.16	0.71	1.40	1172
**ROIC**	75.24 ± 16.38	0.84	1.90	1082
**ROID**	60.00 ± 11.58	0.71	1.40	1166
**ROIE**	69.54 ± 12.27	0.84	1.90	1092

## Data Availability

The data presented in this study are available on request from the corresponding author.
